# A systematic review and meta-analysis of pazopanib efficacy and adverse effects in sarcomas

**DOI:** 10.1186/s12967-026-07775-1

**Published:** 2026-02-02

**Authors:** Fernanda Picozzi, Alessandro Ottaiano, Antonella Lucia Marretta, Mariachiara Santorsola, Lucia Cannella, Antonio Pizzolorusso, Francesco Caraglia, Giuseppina Della Vittoria Scarpati, Alessandra Bracigliano, Ottavia Clemente, Ines Simeone, Michele Caraglia, Salvatore Tafuto

**Affiliations:** 1 Sarcoma and Rare Tumors Unit, Istituto Nazionale Tumori, IRCCS Fondazione “G. Pascale”, 80131 Naples, Italy; 2 Melanoma Unit, Cancer Immunotherapy and Development Therapeutics, Istituto Nazionale Tumori, IRCCS Fondazione “G. Pascale”, 80131 Naples, Italy; 3 SSD Terapie Innovative nelle Metastasi Addominali, Istituto Nazionale Tumori, IRCCS Fondazione “G.Pascale”, 80131 Naples, Italy; 4 Department of Precision Medicine, University of Campania “Luigi Vanvitelli”, 80138 Naples, Italy; 5 Humanitas Research Hospital, 20089 Rozzano, Italy

**Keywords:** Pazopanib, Sarcomas, Response, Toxicity, Meta-analysis

## Abstract

**Background:**

Pazopanib, a multi-targeted tyrosine kinase inhibitor, is employed in the treatment of various malignancies, including metastatic non-adipocytic soft tissue sarcoma.

**Methods:**

This systematic review and meta-analysis adhered to PRISMA guidelines to evaluate the efficacy and toxicity of pazopanib monotherapy in treating sarcomas. A comprehensive search of PubMed/MEDLINE and Scopus/ELSEVIER databases was conducted, covering the period from 2009 to 2025. The included studies were evaluated for quality using the MINORS, Newcastle-Ottawa Scale, and RoB2 tools. The spectrum of toxicities and responses in sarcoma types was described. A meta-analysis was performed to compare the efficacy of pazopanib with non-placebo treatments, using both fixed-effect and random-effect models to calculate pooled hazard ratios (HRs) for progression-free survival (PFS) and overall survival (OS).

**Results:**

A total of 40 studies were included, encompassing a wide range of study designs and quality. The analysis revealed variable objective response rates (ORR) across different sarcoma types, with the highest ORRs by RECIST observed in desmoid tumors (37.0%) and alveolar soft part sarcoma (35.5%). Common toxicities included hypertension, liver function test abnormalities, and fatigue, with significant variability in dose reductions and treatment interruptions across studies. The pooled HRs for PFS and OS were 1.10 (95% CI: 0.69–1.25) and 0.99 (95% CI: 0.63–1.35), respectively, indicating no significant advantage of non-placebo treatments respect to pazopanib.

**Conclusions:**

Pazopanib demonstrated histology-specific efficacy in sarcomas, with a manageable toxicity profile. Furthermore, it does not appear inferior to non-placebo interventions, highlighting the need for further comparative studies to clarify its role in the therapeutic landscape of advanced sarcomas.

**Supplementary Information:**

The online version contains supplementary material available at 10.1186/s12967-026-07775-1.

## Introduction

Pazopanib is an oral multi-targeted tyrosine kinase inhibitor (TKI) with a well-established role in the treatment of various malignancies. As a sulfonamide compound, pazopanib inhibits several key receptor tyrosine kinases (RTKs), including vascular endothelial growth factor receptors (VEGFR-1, VEGFR-2, and VEGFR-3), platelet-derived growth factor receptors (PDGFR-α and PDGFR-β), and fibroblast growth factor receptor (FGFR) [1].

The primary mechanism of action of pazopanib involves the inhibition of angiogenesis, a hallmark of tumor progression and metastasis [2]. VEGF signaling is a major driver of angiogenesis in many cancers, including sarcomas. Pazopanib binds to the intracellular domain of VEGF receptors, blocking their autophosphorylation and thereby inhibiting downstream signaling pathways that promote endothelial cell proliferation and neovascularization [3]. Concurrent inhibition of PDGFR and FGFR enhances its anti-tumor efficacy by disrupting stromal support and impairing tumor cell proliferation [4, 5].

Sarcomas are a heterogeneous group of rare malignancies arising from mesenchymal tissues. Representing approximately 1% of all adult cancers, sarcomas encompass over 90 histological subtypes, contributing to their therapeutic complexity [6]. While surgery remains the cornerstone of treatment for localized disease, the management of metastatic or unresectable sarcomas remains challenging, primarily due to the limited efficacy of conventional cytotoxic chemotherapy. Standard first-line regimens for advanced sarcomas typically include monotherapies or combinations of agents such as anthracyclines, ifosfamide, cisplatin, etoposide, and vincristine. However, objective response rates per RECIST criteria are generally low, and the prognosis remains poor, with median overall survival ranging from 12 to 18 months in metastatic cases [7].

In recent years, pazopanib has emerged as an important therapeutic option for patients with metastatic non-adipocytic soft tissue sarcoma (STS) who have previously received chemotherapy. A pivotal randomized, double-blind, placebo-controlled phase III trial involving 369 patients demonstrated a significant improvement in progression-free survival (PFS) with pazopanib compared to placebo (median PFS: 4.6 months vs. 1.6 months; hazard ratio [HR] = 0.31, 95% confidence interval [CI]: 0.24–0.40, *P* < 0.0001), leading to its regulatory approval in this setting [8]. However, in addition to adipocytic sarcomas, several other histological subtypes were excluded from the trial, including embryonal rhabdomyosarcoma, chondrosarcoma, osteosarcoma, Ewing tumors, primitive neuroectodermal tumors, gastrointestinal stromal tumors (GISTs), dermatofibrosarcoma protuberans, inflammatory myofibroblastic sarcoma, malignant mesothelioma, and mixed Müllerian tumors of the uterus. These exclusions were based on the limited or absent evidence of pazopanib’s efficacy in these histologies at the time of trial design, as well as their distinct biological and molecular profiles compared to other soft tissue sarcomas.

The aim of this study is to provide a comprehensive evaluation of pazopanib monotherapy in patients with sarcomas, specifically assessing its therapeutic activity, as measured by clinical response outcomes, and its safety profile, as defined by the incidence and severity of treatment-related toxicities. Additionally, we evaluate the current literature comparing pazopanib with other active therapeutic alternatives, and assess whether these comparisons translate into meaningful survival benefits. Through this analysis, we aim to contribute to the growing body of evidence on the efficacy and safety of pazopanib, and to further define its role as a single-agent therapy in the treatment landscape for patients with advanced sarcomas.

## Methods

This paper details a systematic review and meta-analysis investigating the toxicity and efficacy of pazopanib in treating sarcomas. The study adheres to the 2020 PRISMA guidelines [9] and was conducted under a meticulously crafted protocol registered with PROSPERO (Prospective Register of Systematic Reviews, managed by the National Institute for Health Research, UK) under CRD42024583953. The protocol outlines in detail the criteria for study selection and the methodologies implemented throughout the review process.

### Search strategy and selection criteria

A comprehensive manual search was carried out in the PubMed/MEDLINE and Scopus/ELSEVIER databases. A manual search strategy was employed to ensure a thorough and context-specific inclusion of studies. To enhance the accuracy and breadth of the search, five researchers were divided into two independent teams (Team 1: F.P., A.L.M, M.S.; Team 2: A.O., S.T.). Team 1 initiated the search, and Team 2 replicated it to ensure consistency and reliability. This dual approach leveraged the varied expertise and perspectives of the researchers, enhancing the sensitivity of the search. Although labor-intensive, this manual strategy allowed for a deeper understanding of the scientific literature, ensuring the inclusion of studies that might be overlooked by automated searches. Each researcher independently reviewed the relevant databases, journals, and supplementary sources, including reference lists of selected articles, to enhance the final dataset. To maintain the integrity of the selection process, the team held regular consensus meetings to resolve any discrepancies that arose.

The search utilized the following keywords: “sarcoma” OR “soft tissue sarcoma” AND “pazopanib” OR “targeted therapy” AND “efficacy” OR “progression-free survival” OR “overall survival” OR “overall response rate.” The literature search spanned from January 2009 to June 2025, utilizing PubMed/MEDLINE and Scopus/ELSEVIER databases known for their comprehensive coverage of biomedical research. The chosen timeframe was carefully selected to include high-quality studies that reflect the most recent advancements in sarcoma treatment, while also capturing relevant historical data.

Specific inclusion criteria were applied to identify relevant studies. Only English-language articles were included, as English is the primary language of scientific communication in clinical oncology, and this criterion is commonly adopted in systematic reviews to ensure consistency, interpretability, and comparability of methodological and outcome data across studies. The review focused on studies involving individuals aged 18 and older with histologically confirmed sarcomas. Eligible studies had to meet the following criteria: (1) evaluate the effect of pazopanib as a single agent, (2) report Hazard Ratios (HRs) with 95% Confidence Intervals (CIs) or response rates (RRs) by RECIST (3) include a sample size of more than five patients, and (4) achieve a score of *≥* 4 on the Newcastle-Ottawa Scale for assessing the quality of non-randomized studies. Regarding response assessment, two studies evaluated response using both RECIST and CHOI criteria. However, only the RECIST-based response was included in our descriptive analyses. Studies that focused solely on prognostic factors affecting pazopanib efficacy were excluded. Studies primarily focused on pharmacokinetic or pharmacodynamic parameters were excluded, as the objective of this review was to evaluate clinical outcomes. Specifically, we did not include studies investigating intra-patient variability in drug plasma concentrations, exposure–response relationships based on therapeutic drug monitoring cohorts, dose–concentration correlations, or pharmacodynamic markers such as target inhibition or modulation of downstream signaling pathways. Data on HRs and RRs were extracted from all sections of the articles, including supplementary materials.

**Fig. 1:**
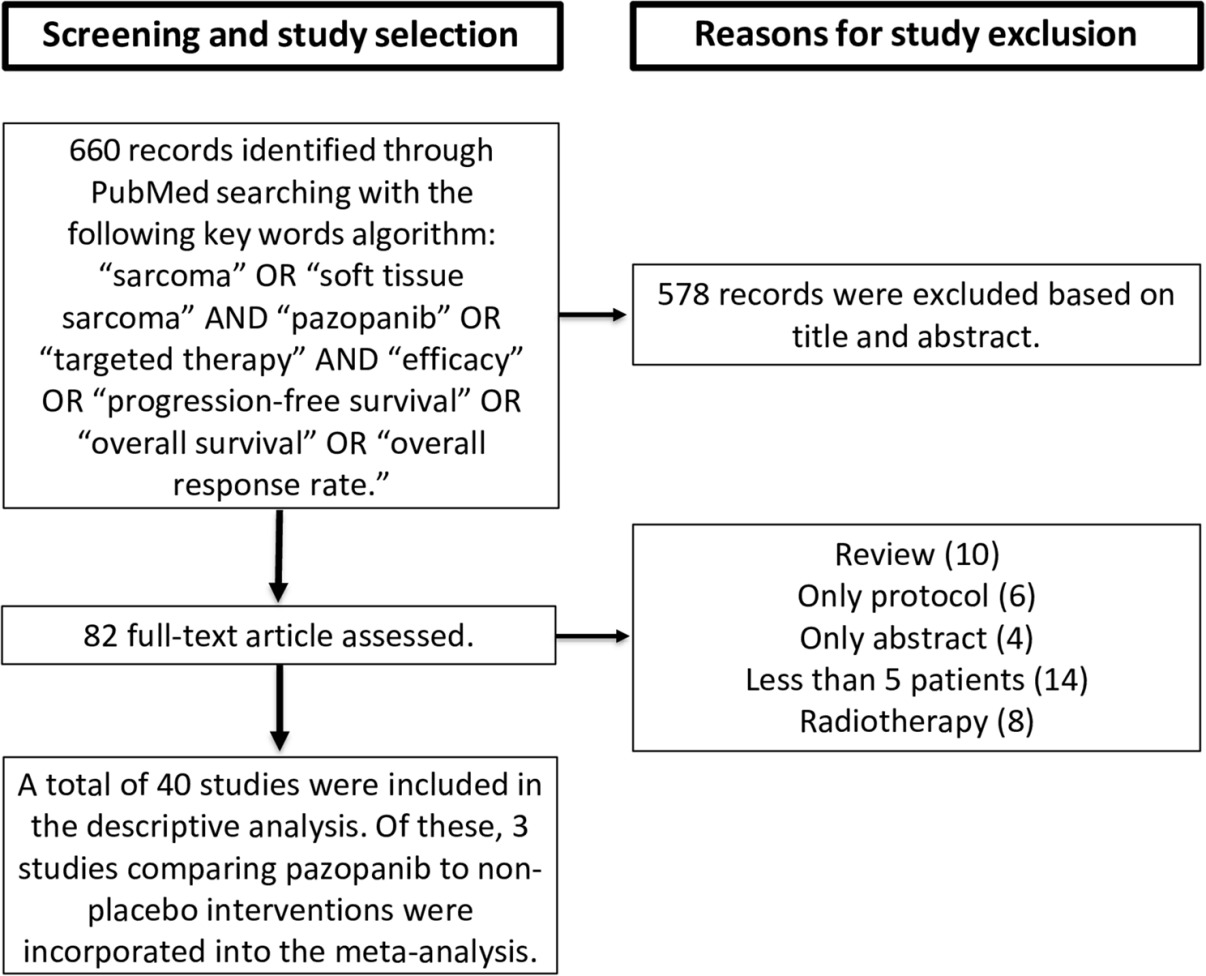
Flowchart illustrating the selection process of studies

There were no restrictions on the study design or specific chemotherapy regimens used as comparator interventions in randomized trials. Preclinical studies that focused exclusively on in vitro or animal experiments were excluded from the analysis. Studies incorporating additional treatments (radiotherapy, biological drugs, surgery) were excluded to ensure a more accurate and focused analysis of pazopanib effects. A detailed flowchart illustrating the study selection process is provided in Fig. 1.

### Data extraction

For each study, we extracted key details including the first author’s name, publication year, patient count, study design information, clinicopathological characteristics (such as age, gender, histotype, objective response by RECIST, line of therapy, toxicity types, and treatment adherence), follow-up data, and hazard ratios (HRs) for progression and/or mortality, along with their 95% confidence intervals (CIs). Any discrepancies or inconsistencies encountered during data extraction were discussed among the research team to reach a consensus.

### Primary objectives

This review and meta-analysis aimed to achieve two main goals: first, to provide a comprehensive evaluation of the responses and toxicities associated with pazopanib in sarcomas, and second, to assess the relative benefits of pazopanib compared to non-placebo interventions.

### Quality assessment

To thoroughly evaluate study quality and potential biases, two separate teams, Team 1 and Team 2, were assigned to assess the methodologies and outcomes of the selected studies. For non-randomized trials, the quality was assessed using the MINORS (Methodological Index for Non-Randomized Studies) [10] and the Newcastle–Ottawa Scale (NOS) criteria [11]. For randomized controlled trials, the Risk of Bias 2 (RoB2) tool [12] was employed. Each team independently rated the studies, and any differences in scoring were resolved through discussion until agreement was reached. The distinct characteristics of the MINORS, NOS, and RoB2 scales, and their respective applications, are elaborated in Supplementary File 1.

### Statistical methods

A meta-analysis was undertaken to evaluate the comparative efficacy of pazopanib against non-placebo treatments. Both fixed-effect and random-effect models were employed, utilizing the DerSimonian and Laird method [13]. In summary, the fixed-effect model operates under the assumption that the true effect size remains consistent across all studies, with variations in observed effect sizes being attributed solely to random fluctuations. Conversely, the random-effect model acknowledges potential differences in the true effect size across studies, attributing observed variations to a combination of random error and genuine differences. Under this model, the true effect size is presumed to vary between studies, and the overall effect is calculated as a weighted average of the individual study effects. This approach often provides a more conservative estimate of the pooled hazard ratio (HR), particularly in the presence of heterogeneity across studies. The results are visually represented using forest plots, which illustrate the hazard ratios (HRs) along with their 95% confidence intervals (CIs) as error bars. The pooled HR estimates are prominently displayed at the end of the graphs.

For clarity and consistency in interpreting the results, it is essential to clarify that the HR compares the likelihood of disease progression or mortality between patients receiving pazopanib and those receiving non-placebo treatments. A HR of 1.0 indicates no difference in risk between the two groups, whereas a HR below 1.0 suggests that pazopanib is associated with a reduced risk of progression or death. Where necessary, HRs were recalculated using Altman’s method to ensure uniformity in the comparative analysis between pazopanib and non-placebo treatments [14].

Study heterogeneity was assessed using the I² statistic [15], which quantifies the percentage of variability across studies attributable to real differences rather than random error. The assessment and interpretation of the I² statistic are reported in Supplementary File 2. Given the high heterogeneity observed among the studies, as indicated by positive results for heterogeneity, no attempts were made to pool the odds ratios for ORR and DCR. In contrast, no such heterogeneity was recorded in studies reporting time-to-outcome.

Publication bias was evaluated using funnel plots [16]. This procedure involves several steps. Initially, effect sizes (HRs) and their corresponding standard errors (SEs) are extracted from the selected studies and plotted on a scatter plot, with effect sizes on the x-axis and precision (SEs) on the y-axis. In the absence of bias, the plot should resemble a symmetrical funnel, where smaller studies are more widely dispersed and larger studies are concentrated towards the top. Deviations from this symmetry may indicate the presence of publication bias, which requires careful consideration of the observed asymmetry’s direction and magnitude. Asymmetry may arise due to selective reporting or methodological inconsistencies. Conceptually, the funnel represents the expected distribution of studies in the absence of bias. Statistical tests, such as Egger’s and Begg’s tests, were applied to formally assess and quantify asymmetry, providing an objective measure of potential publication bias [16]. Detecting asymmetry helps determine whether the overall results might be disproportionately influenced by positive or significant findings. A symmetrical funnel plot suggests a lower probability of publication bias, thereby enhancing the internal validity of the study findings.

All statistical analyses were performed using MedCalc Statistical Software (MedCalc^®^ Statistical Software version 19.6, MedCalc Software Ltd., Ostend, Belgium) and Microsoft Excel^®^ for Windows, version 2302 (Microsoft Corporation, Redmond, WA, USA).

## Results

### Study characteristics

A total of 40 studies were included in the present analysis, encompassing both prospective and retrospective designs, with phases ranging from not reported to phase II and III trials [8, 17–55] (Table 1). The number of patients enrolled varied widely, from as few as 6 patients [26] to as many as 1964 patients [8]. The median follow-up period also exhibited significant variation, ranging from 7 months [39] to 63 months [46].

**Table 1:** Sample size, median follow-up, study design, and quality assessment of selected studies by NOS and MINORS scales

First author	Year	No. of patients	Median follow-up time (months)	Study design	Phase of the study	NOS score	MINORS score
Sleijfer S	2009	142	22.5	Prospective	II	7	12
van de Graaf WTA	2012	369	14.9	Prospective	III	NA	NA
Yoo KH	2015	43	12.0	Retrospective	NR	6	10
Maruzzo M	2015	13	12.3	Prospective	NR	6	9
Nakamura T	2016	156	NR	Retrospective	NR	7	9
Kollàr A	2017	52	15.9	Retrospective	NR	6	6
Jones RL	2017	7	NR	Retrospective	NR	4	5
Kim HJ	2017	35	34	Retrospective	NR	6	8
Stacchiotti S	2018	30	27	Retrospective	NR	6	8
Frezza AM	2018	18	NR	Retrospective	NR	4	5
Kim M	2019	6	33	Prospective	II	4	9
Sunar V	2019	28	10.7	Retrospective	NR	5	10
Stacchiotti S	2019	26	27	Prospective	II	8	13
Martin-Broto J	2019	36	27	Prospective	II	8	13
Toulmonde M	2019	72	23.4	Prospective	II	NA	NA
Seto T	2019	123	NR	Retrospective	NR	7	7
Frezza AM	2019	12	36.3	Retrospective	NR	5	5
Hirbe AC	2020	56	10.83	Prospective	II	5	11
Grunwald V	2020	120	11.8	Prospective	II	NA	NA
Martin-Broto J	2020	34	18	Prospective	II	8	13
Aggerholm-Pedersen N	2020	19	10	Retrospective	NR	6	7
Nishida Y	2020	12	10.6	Retrospective	II	6	8
Oh CR	2020	347	NR	Retrospective	NR	7	9
Chow W	2020	47	41.0	Prospective	II	7	12
Halim NA	2020	15	7	Retrospective	NR	6	7
Liu J	2020	31	35.6	Retrospective	NR	7	10
Schmoll HJ	2021	86	39	Prospective	II	NA	NA
Kataria B	2021	33	18.8	Retrospective	NR	6	7
Park C	2021	24	NR	Retrospective	NR	5	6
Alshamsan B	2021	45	12.4	Retrospective	NR	7	9
Frezza AM	2021	12	15.4	Retrospective	NR	5	7
Jones RL	2022	114	63	Prospective	III	NA	NA
Frankel P	2022	12	14.8	Prospective	II	5	5
Thiebaud JA	2022	29	NR	Prospective	II	6	7
Hong JY	2023	18	40.9	Prospective	NR	6	8
Giani C	2024	36	62.1	Retrospective	NR	7	10
Vincenzi B	2024	1964	51.36	Prospective	NR	6	9
Söylemez CM	2024	81	NR	Retrospective	NR	6	8
Burkhard-Meier A	2025	13	51.4	Retrospective	NR	5	8
Ecker N	2025	99	NR	Retrospective	NR	6	7

MINORS: Methodological Index for Non-Randomized Studies; NOS: Newcastle–Ottawa Scale; NR: Not Reported

Of the 40 studies, 17 were prospective, primarily phase II trials. These prospective studies generally demonstrated robust methodological quality, as indicated by relatively high scores on the NOS and MINORS scales (Table 1). In contrast, 23 studies were retrospective, showing a broader range of methodological quality. For instance, Nakamura T et al. [20] included 156 patients and achieved a NOS score of 7 and a MINORS score of 9, indicating good methodological rigor despite the retrospective design. Conversely, other retrospective studies, such as the one by Jones RL et al. [22] involving 7 patients, had lower NOS and MINORS scores of 4 and 5, respectively, reflecting a less rigorous study design. Overall, the studies analyzed exhibited a broad spectrum of quality and design, with prospective studies generally achieving higher methodological scores and longer follow-up periods compared to retrospective studies. The quality of the included randomized studies was evaluated using the RoB2 tool, and the results are summarized in Table 2. The studies presented varying levels of risk of bias across different domains, with some demonstrating consistently low risks, while others showed moderate to high concerns in specific areas. Among these, four studies reported a comparison between pazopanib and a comparator other than placebo [30, 33, 41, 46].

**Table 2:** Quality assessment of randomized studies through RoB2 tool

First author	Comparator	Year	Randomization process	Deviations from intended intervention	Missing outcome data	Measurement of the outcome	Selection of the reported result	Overall bias
van de Graaf WTA	Placebo	2012	low risk	low risk	low risk	low risk	low risk	low risk
Toulmonde M	Methotrexate-Vinblastine	2019	low risk	some concers	low risk	low risk	some concerns	some concerns
Grunwald V	Doxorubicin	2020	some concerns	low risk	low risk	low risk	some concerns	some concerns
Schmoll HJ	Pazopanib-Gemcitabine	2021	some concerns	low risk	some concerns	low risk	some concerns	some concerns
Jones RL	Pazopanib-Carotuximab	2022	some concerns	low risk	high risk	some concerns	some concerns	high risk

### Clinico-pathological characteristics of enrolled patients

Table 3 provides a summary of the key demographic and clinical characteristics across the selected studies. The patient populations in the pazopanib trials were diverse, varying in age, gender, histology, and line of therapy. The median ages of patients ranged from 26 to 78.7 years, indicating a wide age spectrum. Gender distribution also varied significantly, with the number of male participants ranging from 0 (in studies focused on gynecological sarcomas) to 743, and female participants from 3 to 1221. Histologically, the studies predominantly concentrated on either specific types of sarcoma or a broader array of multiple sarcoma histologies. Only a few studies included osteosarcomas [31, 32]. The line of therapy varied among the studies, with most trials assessing pazopanib in a non-first-line setting, typically involving patients who had already undergone prior treatments.

**Table 3:** Clinico-pathologic characteristics of selected studies

First Author	Year		Median age (years)		Gender			Histology						Line of therapy		
					Male	Female		Single sub-type of STS		Multiple STS		Multiple sarcomas including osteosarcomas		First-line	Other than first	Any
Sleijfer S	2009		51.4		71	71				√						√
van de Graaf WTA	2012		56.7*		99*	147*				√					√	
Yoo KH	2015		54		26	17				√					√	
Maruzzo M	2015		51		6	7		√						√		
Nakamura T	2016		53.8		97	59				√						√
Kollàr A	2017		62.4		32	20				√						√
Jones RL	2017		52		3	4		√								√
Kim HJ	2017		57		0	35				√					√	
Stacchiotti S	2018		33		19	11		√								√
Frezza AM	2018		31		13	5		√								√
Kim M	2019		29.5		3	3		√								√
Sunar V	2019		53		0	28		√							√	
Stacchiotti S	2019		63		21	5		√								√
Martin-Broto J	2019		62		15	21				√						√
Toulmonde M	2019		35*		17*	31*		√								√
Seto T	2019		60		58	65						√				√
Frezza AM	2022		51		7	5		√								√
Hirbe AC	2020		78.7		29	27				√				√		
Grunwald V	2020		71*		44*	37*				√				√		
Martin-Broto J	2020		63		17	17		√								√
Aggerholm-Pedersen N	2020		39		14	5						√				√
Nishida Y	2020		49		6	6		√								√
Oh CR	2020		51		170	177				√					√	
Chow W	2020		58		29	18		√								√
Halim NA	2020		48.6		8	7				√					√	
Liu J	2020		26		10	21		√								√
Schmoll HJ	2021		59*		23*	20*				√					√	
Kataria B	2021		47		18	15				√						√
Park C	2021		NR		NR			√							√	
Alshamsan B	2021		28		23	22				√					√	
Frezza AM	2021		46		4	8		√								√
Jones RL	2022		62.6*		18*	35*		√								√
Frankel P	2022		32		7	5		√**								√
Thiebaud JA	2022		66		12	17		√								√
Hong JY	2023		18		8	10				√						√
Giani C	2024		NR		NR	NR				√						√
Vincenzi B	2024		59.7		743	1221				√						√
Söylemez CM	2024		NR		30	51				√						√
Burkhard-Meier A	2025		43		7	6		√						√		
Ecker N	2025		49.8		46	53				√						√

*These data pertain solely to the pazopanib arm. **Osteosarcoma

### Efficacy of pazopanib

**Fig. 2:**
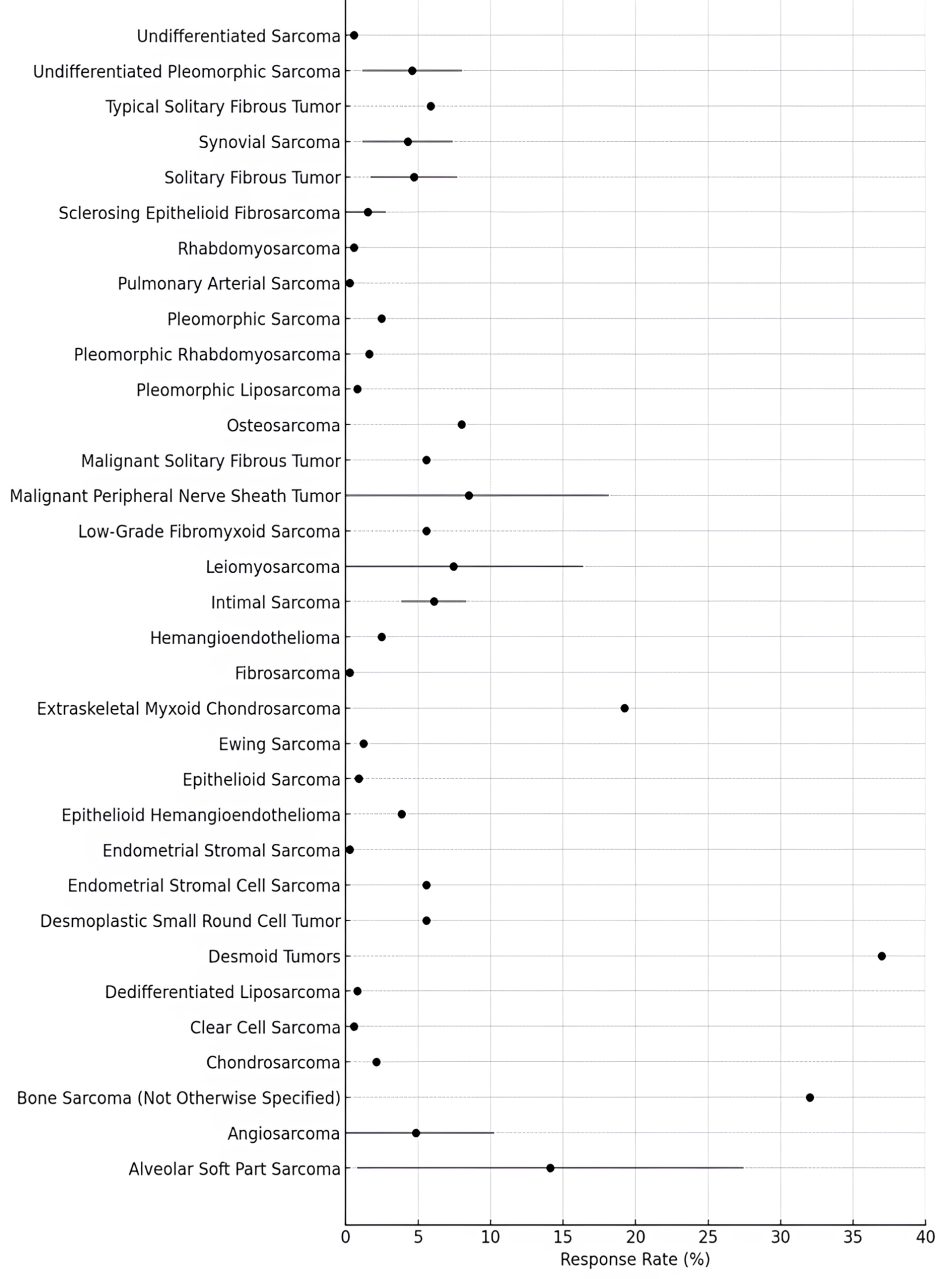
Forest plot showing the mean response rate (RR) for each histotype across studies. Horizontal gray lines represent the standard deviation (SD), where available; for histotypes with only a single data point, only the point estimate is shown. In studies including multiple histotypes, histotype-specific denominators (i.e., the number of responders within a histotype divided by the number of patients with that histotype) were not consistently reported. Therefore, to ensure methodological consistency and enable comparisons across studies, all RRs were calculated using the total study sample size as the denominator. While this approach may underestimate the true histotype-specific RR, particularly for histotypes representing a small fraction of the study population, it offers a conservative and uniform method that facilitates reliable cross-study comparisons

As anticipated, the studies demonstrated variability in ORR by RECIST, disease control rate (DCR), and survival outcomes, including PFS and overall survival (OS) (Table 4). Two studies [29, 34] reported responses evaluated using both RECIST and CHOI criteria. The reported ORR varied widely across the studies, ranging from 0% to 37%. The table lists the histological types in which responses according to RECIST were detected. The highest response rates were reported in desmoid tumors with an ORR of 37% [30] and in alveolar soft part sarcoma (ASPS) with an ORR of 35.5% [40]. Several studies reported no response at all in chondrosarcomas and angiosarcomas [22, 46], in epithelioid sarcomas, epithelioid haemangioendothelioma [25, 45], haemangioendothelioma [54], and in undifferentiated STSs, synovial sarcomas, leiomyosarcomas, angiosarcoma, and desmoid tumor [39], where the ORR was 0%. DCR was inconsistently reported, ranging from 13.3% to 100%. The highest DCR by RECIST was observed in ASPS (100%) [26] and in typical solitary fibrous tumors (100%) [34]. Desmoid tumors also showed a high DCR of 96% [30], while the lowest DCR (13.3%) was observed in undifferentiated STSs and synovial sarcoma [39]. Data on PFS and OS were limited, with HR reported only in a few studies (Table 4). A Forest Plot illustrating the response rates across different histotypes is presented in Fig. 2.

**Table 4:** Detailed outcomes in the selected articles

First author		Year	Sample size		Response to treatment						Progression-free survival				Overall survival		
					**CR** **no(%)**	**Histotype with CR**	ORR (%)*	**Responsive histotypes**	**DCR (%)**		HR**	**CI**	***p***		HR**	**CI**	***p***
Sleijfer S		2009	142		0	-	6.3	LMS, SS	NR		NR	NR	NR		NR	NR	NR
van de Graaf WTA		2012	369		0	-	6	NR	73		0.31	0.24–0.40	< 0.0001		0.86	0.67–1.11	0.2514
Yoo KH		2015	43		0	-	16.3	SS, MFH, UPS, MPNST, LMS, angiosarcoma	60.2		NR	NR	NR		NR	NR	NR
Maruzzo M		2015	13		0	-	9	Solitary fibrous tumours	82		NR	NR	NR		NR	NR	NR
Nakamura T		2016	156		0	-	8.3	UPS, SS, ASPS	55.7		NR	NR	NR		NR	NR	NR
Kollàr A		2017	52		1(1.9)	HE	23.1	Angiosarcoma, epithelioid HE, intimal sarcoma	44.3		NR	NR	NR		NR	NR	NR
Jones RL		2017	7		0	-	0	None	71.4		NR	NR	NR		NR	NR	NR
Kim HJ		2017	35		1(3)	LMS	29	LMS	60		NR	NR	NR		NR	NR	NR
Stacchiotti S		2018	30		1(3.3)	ASPS	27	ASPS	83		NR	NR	NR		NR	NR	NR
Frezza AM		2018	18		0	-	0	None	50		NR	NR	NR		NR	NR	NR
Kim M		2019	6		0	-	16.7	ASPS	100		NR	NR	NR		NR	NR	NR
Sunar V		2019	28		0	-	14.3	LMS	75		NR	NR	NR		NR	NR	NR
Stacchiotti S		2019	26		0	-	18	Extraskeletal myxoid chondrosarcoma	91		NR	NR	NR		NR	NR	NR
Martin-Broto J		2019	36		0	-	6	Malignant solitary fibrous tumour	66		NR	NR	NR		NR	NR	NR
Toulmonde M		2019	72		0	-	37	Desmoid tumours	96		NR	NR	NR		NR	NR	NR
Seto T		2019	123		1(0.8)	LGFMS	10.6	Angiosarcoma, dedifferentiated liposarcoma, LMS, pleomorphic liposarcoma, pleomorphic rhabdomyosarcoma, LGFMS	38.2		NR	NR	NR		NR	NR	NR
Frezza AM		2019	12				8	Intimal sarcoma	42		NR	NR	NR		NR	NR	NR
Hirbe AC		2020	56		1(1.8)	LMS	8.9	UPS, SS, LMS	39.2		NR	NR	NR		NR	NR	NR
Grunwald V		2020	120		1(1.2)	NR	12.3	NR	62.9		1.00	0.65–1.53	NR		1.08	0.68–1.72	0.735
Martin-Broto J		2020	34		0	-	6	Typical solitary fibrous tumours	100		NR	NR	NR		NR	NR	NR
Aggerholm-Pedersen N		2020	19		0	-	32	Bone sarcoma (not otherwise specified)	69		NR	NR	NR		NR	NR	NR
Nishida Y		2020	12		0	-	25	MPNST	50		NR	NR	NR		NR	NR	NR
Oh CR		2020	347		0	-	15.6	LMS, UPS, angiosarcoma, SS, MPNST, solitary fibrous tumor, ASPS, rhabdomyosarcoma, epithelioid sarcoma, endometrial stromal sarcoma, pulmonary arterial sarcoma, clear cell sarcoma, sclerosing epithelioid fibrosarcoma, fibrosarcoma	54.8		NR	NR	NR		NR	NR	NR
Chow W		2020	47		0	-	2	Chondrosarcoma	66		NR	NR	NR		NR	NR	NR
Halim NA		2020	15		0	-	0	None	13.3		NR	NR	NR		NR	NR	NR
Liu J		2020	31		1(3.2)	ASPS	35.5	ASPS	90.3		NR	NR	NR		NR	NR	NR
Schmoll HJ		2021	86		0	-	5	NR	47		0.58	0.36–0.92	0.02		0.98	0.60–1.58	0.83
Kataria B		2021	33		0	-	27	NR	70		NR	NR	NR		NR	NR	NR
Park C		2021	24		0	-	16.7	Angiosarcoma	NR		NR	NR	NR		NR	NR	NR
Alshamsan B		2021	45		0	-	20	NR	55.5		NR	NR	NR		NR	NR	NR
Frezza AM		2021	12		0	-	0	None	25		NR	NR	NR		NR	NR	NR
Jones RL		2022	114		0	-	13	NR	NR		0.98	0.52–1.84	0.95		0.79	0.41–1.51	0.47
Frankel P		2022	12		0	-	8	Osteosarcoma	50		NR	NR	NR		NR	NR	NR
Thiebaud JA		2022	29		0	-	3	Angiosarcoma	48		NR	NR	NR		NR	NR	NR
Hong JY		2023	18		0	-	12.5	LMS, SS	62.5		NR	NR	NR		NR	NR	NR
Giani C		2024	36		0	-	33	LGFMS, SEF, Hybrid-LGFMS/SEF	58.3		NR	NR	NR		NR	NR	NR
Vincenzi B		2024	1964		0	-	NR	LMS, UPS, SS, angiosarcoma, malignant solitary fibrous tumor, MPNST, fibrosarcoma, myxofibrosarcoma, epithelioid sarcoma, pleomorphic rhabdomyosarcoma, MFH, epithelioid HE, DSRCT, clear cell sarcoma, ASPS, other (not specified)	NR		NR	NR	NR		NR	NR	NR
Söylemez CM		2024	81		0	-	18.8	SS, pleomorphic sarcoma, LMS, ASPS, MPNST, fibrosarcoma, angiosarcoma, chondrosarcoma, Ewing sarcoma, epitheloid sarcoma, HE	46.3		NR	NR	NR		NR	NR	NR
Burkhard-Meier A		2025	13		0	-	0	HE	85		NR	NR	NR		NR	NR	NR
Ecker N		2025	99		0	-	14	LMS, SS, myxofibrosarcoma, fibrosarcoma, liposarcoma, UPS, myofibroblastic sarcoma, pleiomorphic sarcoma, rhabdomyosarcoma, angiosarcoma, clear cell sarcoma, epithlioid sarcoma, epithelioid HE, follicular dendritic cell sarcoma, MPNST, spindle cell sarcoma, undifferentiated uterus sarcoma.	40.45		NR	NR	NR		NR	NR	NR

ASPS: Alveolar Soft Part Sarcoma; CR: complete response; HE: hemangioendothelioma; LGFMS: Low-Grade FibroMyxoid Sarcoma; LMS: Leiomyosarcoma; MFH: Malignant Fibrous Histiocytoma; SS: Synovial Sarcoma; MPNST: Malignant Peripheral Nerve Sheath Tumor; SEF: Sclerosing Epithelioid Fibrosarcoma; UPS: Undifferentiated Pleomorphic Sarcoma. *(Complete and Partial Responses) According to RECIST v1.1 criteria: **HRs as reported in comparative trials were considered (studies reporting HRs derived solely from prognostic models, multivariate analyses, or subgroup analyses were excluded)

### Toxicity and compliance to pazopanib

A comprehensive analysis of toxicities associated with various treatments across multiple studies was performed, focusing on dose reductions, treatment interruptions, and the most common grade III-IV toxicities. The results are summarized in Table 5. The proportion of patients who required dose reductions due to toxicity varied significantly from 23% [17, 19] to 60% [46]. The incidence of treatment interruptions due to toxicity also exhibited considerable variability. Notably, Frezza et al. [49] reported a 100% treatment interruption rate, with other high rates observed in Jones et al. [46] at 94% and Nishida et al. [36] at 66.6%. However, these studies did not clearly distinguish the causes of treatment interruptions, such as disease progression, toxicity, patient choice, or other factors. In contrast, significantly lower interruption rates were reported by Burkhard-Meier et al. [54], Martin-Broto et al. [29], and Sleijfer et al. [17] at 0%, 3%, and 6%, respectively. The median duration of treatment also varied across studies. The longest median treatment duration was observed at 12 cycles in ASPS [24], and at 16.4 weeks in advanced non-adipocitic STS [8]. The most common grade III-IV toxicities observed across the studies demonstrated significant variability. Hypertension was the most frequently reported toxicity, followed by liver function test abnormalities (LFTA). Other commonly observed toxicities included diarrhea, neutropenia, and fatigue. Some studies [30, 38] lacked sufficient data on dose reductions or treatment interruptions, highlighting the heterogeneity in reporting across trials.5

**Table 5:** Toxicity and compliance associated with pazopanib in the analyzed studies

First author	Year	% of patients who experienced dose-reduction for toxicity	% of patients who experienced drug interruption for toxicity	Median duration of treatment	Type of three most common grade III-IV toxicities
Sleijfer S	2009	23	6	NR	Hepatotoxicity; Cardiac toxicity
van de Graaf WTA*	2012	39	49**	16.4 weeks	Hypertension; Cardiac toxicity; Elevated bilirubin
Yoo KH	2015	48.8	2.3	4.8 cycles	Stomatitis; Anemia; Diarrhea
Maruzzo M	2015	23	15	4.1 months	LFTA; Hypertension; Hyponatraemia
Nakamura T	2016	NR	28.2	28.7 weks	NR
Kollàr A	2017	NR	NR	NR	LFTA; Fatigue; Neutropenia
Jones RL	2017	NR	NR	NR	Fatigue; LFTA; Elevated blood pressure
Kim HJ	2017	NR	80**	4 cycles	Hypertension; Transaminases elevation; Neutropenia
Stacchiotti S	2018	NR	13.3	12 cycles	Hand-foot syndrome; Diarrhea; Anorexia
Frezza AM	2018	NR	NR	2 cycles	NR
Kim M	2019	NR	NR	7.5 cycles	Fatigue; Hypertension; Anorexia
Sunar V	2019	NR	7.1	5 months	NR
Stacchiotti S	2019	30	9	15 cycles	NR
Martin-Broto J	2019	28	3	5.5 cycles	NR
Toulmonde M*	2019	NR	8	NR	NR
Seto T	2019	NR	NR	3 months	Mucositis; Hand-foot syndrome; Deep vein thrombosis
Frezza AM	2019	NR	100**	NR	LFTA; Hypertension; Neutropenia
Hirbe AC	2020	19.6	9	NR	Hypertension; LFTA; Diarrhea
Grunwald V*	2020	24.7	32.1	3.8 months	Hypertension; Diarrhea; Fatigue
Martin-Broto J	2020	52	84**	8 cycles	Diarrhea
Aggerholm-Pedersen N	2020	26	10	NR	NR
Nishida Y	2020	50	66.6**	NR	Hypertension; Hand-foot syndrome; Diarrhea
Oh CR	2020	32.6	NR	NR	Hypertension; Fatigue; Thrombocytopenia
Chow W	2020	NR	26	NR	NR
Halim NA	2020	33	26.7	3 months	Hypertension; LFTA; Hyponatremia
Liu J	2020	35.5	NR	NR	Hypertension; LFTA; Diarrhea
Schmoll HJ*	2021	NR	NR	NR	LFTA; WBC decrease; Cardiac toxicity
Kataria B	2021	12	21	NR	Diarrhea; Nausea; Vomiting
Park C	2021	NR	NR	NR	LFTA; Hypertension; Infection
Alshamsan B	2021	31	6	NR	NR
Frezza AM	2021	NR	91**	NR	LFTA
Jones RL*	2022	60	94**	NR	Hypertension; Pneumothorax; Liver disorder
Frankel P	2022	NR	17	NR	LFTA; Hypertension; Neutropenia
Thiebaud JA	2022	10	14	2 cycles	Hypertension; Diarrhea; Neutropenia
Hong JY	2023	NR	NR	NR	NR
Giani C	2024	NR	NR	4 cycles	NR
Vincenzi B	2024	25.2	9.6	3 cycles	NR
Söylemez CM	2024	6.1	1.5	NR	Fatigue; Arrhythmia; Pneumothorax
Burkhard-Meier A	2025	23	0	8.8 months	Fatigue; Hypothyroidism; Hair color changes
Ecker N	2025	39.4	17.2	NR	NR (Grade 3 toxicities are grouped with Grade 2; Grade 4 not reported)

*The data refer solely to the pazopanib arm. **In these studies, there is no clear distinction among the causes of treatment interruptions, such as disease progression, toxicity, patient choice, or other factors. LFTA: Liver Function Test Abnormalities

### Comparative efficacy of pazopanib versus active non-placebo interventions

**Fig. 3:**
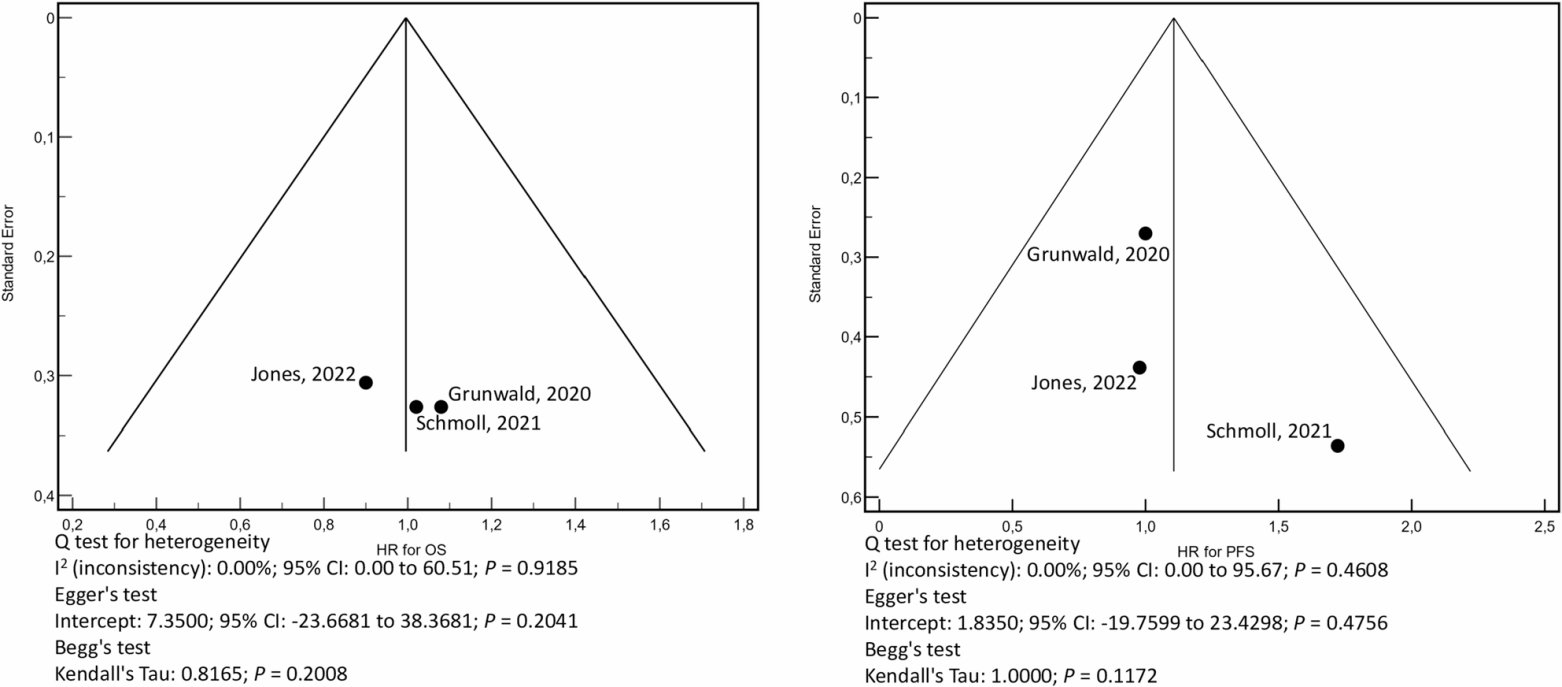
The left funnel plot depicts the hazard ratios (HR) for Overall Survival (OS), and the right plot shows the hazard ratios (HR) for Progression-Free Survival (PFS). In each plot, individual studies are represented as points plotted against their standard error, with the HR values on the x-axis. Symmetry around the central line indicates the absence of publication bias. Below the plots, results from the Q test, Begg’s test, and Egger’s test are shown. No significant asymmetry is observed in either plot, and all tests for heterogeneity are non-significant, confirming the absence of publication bias

**Fig. 4:**
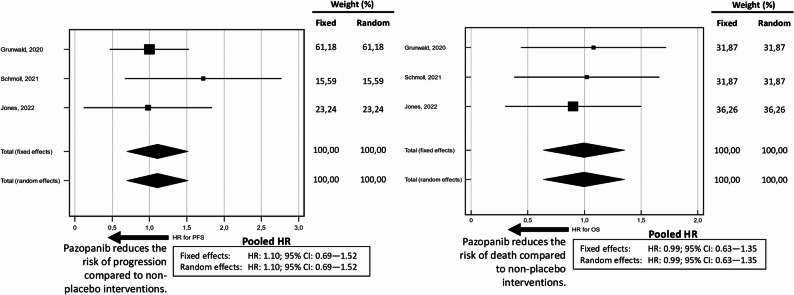
Forest plots displaying the pooled hazard ratios (HRs) for Progression-Free Survival (PFS) on the left and Overall Survival (OS) on the right, analyzed using both fixed-effects and random-effects models. The weight of each study is indicated alongside the corresponding plot. Arrows indicate the direction of effect, showing a trend toward risk reduction in favor of pazopanib compared to non-placebo interventions

To determine whether pazopanib offers advantages over other active treatment options, three studies were selected for meta-analysis, assessing the efficacy of pazopanib against non-placebo interventions in various sarcoma types [33, 41, 46]. These studies included a total of 320 patients in the progression-free survival and overall survival analyses. No significant heterogeneity was observed, and the funnel plots indicated a symmetrical distribution among the selected studies (Fig. 3). The Forest Plots for PFS and OS are presented in Fig. 4. The pooled HR for PFS and OS were 1.10 (95% CI: 0.69–1.25) and 0.99 (95% CI: 0.63–1.35), respectively, consistent across both fixed and random-effects models. Notably, these estimates showed similar efficacy compared to alternative interventions considered active to date. (Table 2).

## Discussion

The results of this systematic review and meta-analysis provide an overview of the efficacy and toxicity of pazopanib in treating sarcomas.

Pazopanib has shown variable efficacy across STS subtypes and treatment settings. Its clinical utility appears greatest in specific histologies such as desmoid tumors and ASPS, where high ORRs and DCRs were consistently observed, up to 37% and 35.5%, respectively. Although rare, complete responses were primarily reported in leiomyosarcoma and ASPS, further supporting the notion that pazopanib, despite its approval for all non-adipocytic STSs, offers greater benefit in selected subtypes. Importantly, responses were also reported in challenging histologies such as malignant peripheral nerve sheath tumors and extraskeletal myxoid chondrosarcoma (Table 4). In contrast, limited or no activity was observed in chondrosarcoma, epithelioid sarcoma, and undifferentiated sarcomas, where ORRs were consistently 0%. Across studies, pazopanib was predominantly evaluated in pre-treated patients, typically in the second-line setting or beyond. Key limitations include heterogeneity in study design, absence of histotype-specific denominators in most reports, inconsistent documentation of DCR and toxicity, and a lack of superiority over other active treatments in comparative trials. Importantly, these negative or modest outcomes should not be uniformly interpreted as evidence of intrinsic biological resistance, but may in part reflect methodological factors that underestimate therapeutic benefit. Collectively, these findings support the use of pazopanib as a relevant option in select STS subtypes, while highlighting its limited or uncertain role in others, emphasizing the need for more histology-driven, prospective investigations.

Assessing radiologic response and, consequently, activity of pazopanib in sarcomas is challenging. Only two studies in this analysis evaluated response using both RECIST and CHOI criteria, while the remaining studies employed RECIST alone. This reliance on RECIST, which primarily captures tumor size reduction, may systematically underestimate the activity of antiangiogenic agents such as pazopanib, as these therapies often induce changes in tumor density, necrosis, or vascularization rather than rapid dimensional shrinkage. In contrast, CHOI criteria account for these changes, offering potential advantages in detecting therapeutic response, particularly in sarcomas, where size changes may not be the sole indicator of treatment efficacy and failure [56, 57]. Thus, some cases of apparent “non-response” may reflect limitations of response assessment rather than true lack of biological activity.

In terms of toxicity, pazopanib was associated with a range of adverse effects, with hypertension being the most frequent, followed by liver function abnormalities, diarrhea, and fatigue. These toxicities, while highlighting the need for careful management and monitoring during treatment to minimize adverse effects, are generally manageable. This is consistent with the existing evidence in scientific literature [58–60].

Placebo-controlled designs are generally considered ethically acceptable when no effective standard therapies are available. However, the expanding landscape of alternative therapeutic options necessitates careful reconsideration of this approach. It is therefore increasingly important to determine whether pazopanib, approved for use in non-adipocytic soft tissue sarcomas from the second-line setting, provides efficacy advantages not only over placebo but also in comparison with other active treatment strategies. In this context, the pooled hazard ratios for progression-free survival and overall survival did not demonstrate a significant advantage of non-placebo interventions over pazopanib. It is noteworthy that pazopanib was not inferior to more aggressive chemotherapy regimens, such as methotrexate-vinblastine in desmoid tumors [30] and doxorubicin in diverse sarcomas [33]. The response rates observed with pazopanib are notably significant, particularly when considering its use beyond the first-line of treatment. This significance is underscored when comparing pazopanib to the outcomes of large randomized studies focused on first-line therapies. In these studies, doxorubicin monotherapy typically yields response rates of around 15%. When combined with ifosfamide, response rates increase to approximately 30%. However, the combination of anthracyclines and ifosfamide is also associated with considerable severe toxicities, including myelosuppression, dose-dependent cardiotoxicity, and mucositis [61, 62]. Notably, the absence of superiority should be interpreted in light of late-line treatment settings, where disease biology and prior resistance mechanisms may confound efficacy estimates and bias results toward underestimation of benefit.

The heterogeneous response to pazopanib across histological subtypes of STS represents a critical challenge highlighted by this study. While the drug shows notable clinical efficacy in specific subtypes such as leiomyosarcoma and alveolar soft part sarcoma, many others (including undifferentiated pleomorphic sarcoma and liposarcoma) typically demonstrate only modest benefit. This variability likely reflects the profound biological and molecular heterogeneity that characterizes STSs [63–65]. Each sarcoma subtype harbors distinct oncogenic drivers, patterns of vascularization, and stromal microenvironments that can influence sensitivity to pazopanib. For example, alveolar soft part sarcoma is marked by a high degree of vascularization and a characteristic *ASPSCR1*-*TFE3* fusion, potentially rendering it particularly susceptible to VEGF-targeted agents [66]. In contrast, liposarcomas (especially the well-differentiated and dedifferentiated forms) are generally less vascularized and may not depend heavily on angiogenic pathways, possibly accounting for their reduced responsiveness to pazopanib [67]. Another important limitation contributing to the observed variability in clinical outcomes is the design of many trials, which often do not include stratification based on molecular subgroups. The frequent lack of molecular stratification in clinical trials further complicates interpretation, as biologically distinct entities are often analyzed together, potentially obscuring subtype-specific efficacy signals. A key example is the frequent omission of the distinction between translocation-associated and non-translocation STSs, despite their well-documented differences in molecular architecture and drug sensitivity; this lack of molecular stratification may mask subtype-specific efficacy signals [68].

Taken together, these considerations highlight that the observed heterogeneity in clinical outcomes likely arises from an interplay between true biological resistance and methodological constraints inherent to trial design, response assessment, and patient selection. These considerations highlight the need for innovative and pragmatic trial designs to more accurately assess comparative efficacy in rare and heterogeneous sarcoma populations. Future randomized studies may benefit from basket trial approaches, histology-agnostic but biomarker-driven designs, or the incorporation of well-defined real-world comparators, which could enhance feasibility while preserving clinical relevance. Such strategies may allow more precise identification of patient subsets most likely to benefit from pazopanib and better contextualize its role relative to emerging therapeutic options. To overcome these challenges, future research should first and foremost prioritize the refinement of patient selection through the identification and validation of novel biomarkers predictive of response to pazopanib. Although the evidence is still preliminary, several promising avenues include the use of systemic inflammatory indices (e.g., neutrophil-to-lymphocyte ratio [NLR], platelet-to-lymphocyte ratio [PLR]) [69, 70], the presence of *PDGFRA* mutations [71], and co-amplifications of *PDGFRA*, *VEGFR2*, and *KIT* [72]. These markers could help establish a molecular rationale for more individualized treatment approaches, ultimately enhancing therapeutic precision and optimizing outcomes in patients most likely to benefit from pazopanib.

Recently, cabozantinib has emerged as a compelling alternative to pazopanib for the treatment of soft tissue and bone sarcomas [73]. Like pazopanib, it is a multi-kinase inhibitor, but it has a broader target profile that includes MET and AXL, which are implicated in tumor invasiveness and resistance mechanisms. This expanded activity spectrum may offer particular advantages in sarcoma subtypes driven by MET signaling, such as osteosarcoma and Ewing sarcoma, which are often refractory to VEGFR inhibitors [74]. In the CABONE phase 2 trial, cabozantinib demonstrated encouraging clinical activity in these settings, with median progression-free survival ranging from 4 to 6 months and disease control rates approaching 60% [75]. Both pazopanib and cabozantinib are orally administered once daily, yet their safety profiles differ. In addition to class-related toxicities such as fatigue, hypertension, and QT prolongation, cabozantinib more frequently induces adverse effects like palmar–plantar erythrodysesthesia and mucositis.

Importantly, both agents have also been evaluated in combination with immune checkpoint inhibitors (ICIs) in STS [76]. This therapeutic strategy is particularly intriguing given their ability to modulate the tumor microenvironment and potentially enhance antitumor immunity. Pazopanib has been shown to attenuate immunosuppressive angiogenic signaling, thereby promoting T-cell infiltration and function [77]. Similarly, cabozantinib has demonstrated immunomodulatory effects, including the reduction of myeloid-derived suppressor cells and regulatory T cells [78]. These properties provide a compelling rationale for combining these agents with PD-1 or CTLA-4 blockade. In a retrospective study, a subset of patients received pazopanib plus nivolumab, with evidence of partial response, especially in histotypes typically less responsive to pazopanib (dedifferentiate chondrosarcoma and epithelioid sarcoma) [79]. In a phase 2 study, the combination of pazopanib with the PD-L1 immune checkpoint inhibitor durvalumab demonstrated unexpectedly meaningful clinical activity in STS subtypes that typically show limited responsiveness to pazopanib monotherapy (desmoplastic small round cell tumour, undifferentiated pleomorphic sarcoma, and malignant peripheral nerve sheath tumour). The overall response rate of 30.4% surpassed the predefined efficacy threshold, and a median PFS of 7.7 months was achieved. Crucially, high intratumoral infiltration by CD20 + B cells, along with increased vessel density, emerged as independent predictors of prolonged PFS and superior clinical outcomes [80]. These findings suggest that the tumor immune microenvironment may play a critical role in mediating response to this therapeutic combination. In line with this synergistic rationale, results from a randomized phase II trial demonstrated that the combination of cabozantinib with dual immune checkpoint inhibition (nivolumab and ipilimumab) significantly improved disease control and PFS in patients with metastatic STS, particularly in leiomyosarcoma. Notably, responses were also observed in angiosarcoma, epithelioid sarcoma, and myxofibrosarcoma [81]. Together, these studies support the emerging paradigm that integrating antiangiogenic agents with immunotherapy may enhance clinical benefit across a broader spectrum of STS subtypes than previously recognized, and underscore the need for biomarker-driven strategies to guide therapeutic decision-making.

Our study has several limitations. The first stems from the methodological heterogeneity of the included studies, which varied widely in design (encompassing both prospective and retrospective approaches) and in sample size, quality scores, and follow-up duration. However, this heterogeneity is multifaceted. For instance, the diversity in patient populations, reflected by a wide age range (26 to 78.7 years) and varying gender distributions, underscores the inherently heterogeneous nature of sarcomas. Furthermore, the inclusion of different histological types, ranging from STSs to osteosarcomas, complicates the evaluation of pazopanib’s efficacy across such a diverse spectrum of tumors. In this context, predefined subgroup analyses by histologic subtype were neither planned nor feasible, given the marked granularity of sarcoma histotypes and the limited number of patients within individual subgroups, which would not have allowed statistically robust or clinically meaningful comparisons. Additional complexity arises from variability in the line of therapy at which pazopanib was administered. Most studies evaluated pazopanib in non-first-line settings (Table 3), a scenario that mirrors real-world clinical practice. However, this approach may underestimate the true efficacy of pazopanib in non-randomized settings, as the drug is often offered to patients with more advanced disease or following prior treatment failures. As a result, the potential therapeutic benefit may be confounded by the disease’s progression stage at the time of treatment initiation.

Second, although the hazard ratios for PFS and OS suggest that pazopanib may be a viable alternative to more intensive therapeutic strategies, the limited availability of time-to-outcome data (combined with substantial heterogeneities) limits the strength of any conclusions regarding its long-term efficacy. Therefore, even in the absence of significant statistical heterogeneity, the paucity of data warrants caution in interpreting the pooled HRs.

A third limitation involves methodological variability in toxicity assessment. Notably, discrepancies in dose reduction and treatment interruption rates likely reflect not only true differences in pazopanib tolerability across histological subtypes but also variability in treatment protocols and adverse event reporting practices. High rates of treatment discontinuation reported in some studies [46, 49] were influenced by factors beyond toxicity, including disease progression or patient preference. Future studies should clearly differentiate the reasons for treatment interruption to better assess the role of toxicity in treatment adherence.

Our results underscore that pazopanib is not uniformly effective across STS subtypes, despite its broad regulatory approval. Its most meaningful benefit appears confined to select histologies where both ORRs and DCRs are consistently high. This suggests that pazopanib should not be considered a default option for all non-adipocytic sarcomas, but rather integrated into histology-driven treatment algorithms. Clinically, this means reserving pazopanib for subtypes where angiogenesis plays a central role or where other systemic options are limited. Furthermore, our findings emphasize that pazopanib’s efficacy in pretreated populations compares favorably to other non-placebo interventions, despite its oral administration and generally manageable toxicity. This is particularly relevant in frail patients or those for whom cytotoxic chemotherapy is contraindicated. Thus, pazopanib serves not only as a bridging therapy but also as a viable alternative in specific second- or third-line settings. However, the variability in radiologic response and toxicity reporting across studies underscores the need for standardization in evaluating targeted therapies. The limited use of CHOI criteria, for example, may have led to underestimation of benefit in certain subtypes. Clinicians should be aware that traditional RECIST assessments might not fully reflect biological activity, particularly in vascular sarcomas. Finally, the integration of pazopanib with immunotherapy, and its potential predictive markers such as CD20 + B cell infiltration, suggests a future paradigm shift toward combination strategies. This approach holds promise for expanding pazopanib’s utility beyond traditionally responsive subtypes and warrants further clinical exploration.

## Conclusions

Pazopanib remains an important therapeutic option for previously treated, non-adipocytic soft tissue sarcomas, offering consistent disease stabilization across several histologies despite modest objective response rates. Its efficacy appears particularly notable in selected subtypes such as alveolar soft part sarcoma, desmoid tumors, and solitary fibrous tumors. Toxicity is frequent and often necessitates dose modifications, underscoring the need for careful clinical monitoring and optimized management strategies. Further research is essential to identify specific patient subgroups that may derive greater benefit from pazopanib and to explore potential biomarkers that could predict treatment response. This continued investigation will be crucial for refining therapeutic allocation, improving risk–benefit balance, and ultimately enhancing outcomes for patients with advanced sarcomas.

## Supplementary Information

Below is the link to the electronic supplementary material.

Supplementary Material 1

Supplementary Material 2

## Data Availability

The datasets used and/or analyzed during the current study are available from the corresponding author on reasonable request.

## Abbreviations

TKI: Tyrosine kinase inhibitor
RTK: Receptor tyrosine kinase
VEGFR: Vascular endothelial growth factor receptor
PDGFR: Platelet-derived growth factor receptor
FGFR: Fibroblast growth factor receptor
VEGF: Vascular endothelial growth factor
RECIST: Response evaluation criteria in solid tumors
PFS: Progression-free survival
HR: Hazard ratio
CI: Confidence interval
GIST: Gastrointestinal stromal tumor
STS: Soft tissue sarcoma
PRISMA: Preferred reporting items for systematic reviews and meta-analyses
PROSPERO: Prospective register of systematic reviews
RR: Response rate
CHOI: Choi response criteria
MINORS: Methodological index for non-randomized studies
NOS: Newcastle–ottawa scale
RoB2: Risk of bias 2 tool
I²: I-squared statistic (measure of heterogeneity)
Q: Cochran’s heterogeneity statistic
DF: Degrees of Freedom
ORR: Overall response rate
DCR: Disease control rate
LFTA: Liver function test abnormalities
ASPS: Alveolar soft part sarcoma
OS: Overall survival
SE: Standard error
NLR: Neutrophil-to-lymphocyte ratio
PLR: Platelet-to-lymphocyte ratio
PDGFRA: Platelet-derived growth factor receptor alpha (gene)
VEGFR2: Vascular endothelial growth factor receptor 2
KIT: KIT proto-oncogene receptor tyrosine kinase
MET: MET proto-oncogene (mesenchymal–epithelial transition factor)
AXL: AXL receptor tyrosine kinase
ICIs: Immune checkpoint inhibitors
PD-1: Programmed cell death protein 1 (PD-1)
CTLA-4: Cytotoxic T-lymphocyte associated protein 4 (CTLA-4)
PD-L1: Programmed death-ligand 1 (PD-L1)
CD20: Cluster of differentiation 20 (B-cell marker)
ASPSCR1-TFE3: ASPSCR1–TFE3 gene fusion
