# Supplementary material for: A systematic review and meta-analysis of pazopanib efficacy and adverse effects in sarcomas
Source: J Transl Med. 2026 Feb 2;24:311. doi: 10.1186/s12967-026-07775-1 (PMC12955024; doi:10.1186/s12967-026-07775-1)
Supplement: Supplementary file 1 — Supplementary Material 1 [file 12967_2026_7775_MOESM1_ESM.doc]

Supplementary File 1

Various methodological aspects and outputs of the quality assessment scales employed in the study.

| **Aspect evaluated** | **MINORS** | **NOS** | **RoB2** |
| --- | --- | --- | --- |
| Study design | Explicit criteria for assessing non-  Randomized studies | Differentiates between cohort and case-control  studies | Appraises the  Randomization process and study design |
| Control group | Presence and  appropriateness of a control group | Assesses the selection  and comparability of the control group | Evaluates allocation  concealment, blinding, and baseline similarities |
| Baseline comparability | Evaluates baseline  comparability between groups | Examines comparability based on characteristics  and confounders | Assesses baseline similarities and  adjustments |
| Patient selection | Clear description of patient selection process | Criteria for patient selection and  Representativeness of  the sample | Evaluates the  Recruitment process and eligibility criteria |
| Endpoint assessment | Adequate reporting and evaluation of study endpoints | Assess the outcome measurement and ascertainment of  outcomes | Evaluates outcome measurement and reporting |
| Statistical analysis | Considers statistical analysis and power calculation | Considers statistical methods,  appropriateness, and power | Examines statistical analysis, bias, and precision |
| Loss to follow-up | Addresses and  accounts for loss to follow-up | Considers loss to  follow-up and adequacy of follow-up duration | Evaluates completeness of follow-up and  Handling of missing data |
| Confounding factors | Assesses the control of confounding factors | Addresses confounding factors and control  measures | Examines methods to  Control confounding and other biases |
| Interventions/Exposures | Evaluates the description and appropriateness | Examines exposure/intervention definition and  measurement | Assesses  intervention/exposure, randomization, and  blinding |
| Quality of reporting | Considers the overall quality of reporting | Examines the clarity  and completeness of reporting | Assesses reporting bias  and selective outcome reporting |
| Score range | 0 -16 | 0-9 (for cohort or case-  control studies),0-10 (for cohort studies) | 0 -11 |
| Main aspect described | Assesses the overall methodologicalquality of non-randomized  studies | Focuses on selection, comparability, and  outcome assessment for observational studies | Evaluates the risk of bias related to study design |
