# Supplementary material for: A systematic review and meta-analysis of pazopanib efficacy and adverse effects in sarcomas
Source: J Transl Med. 2026 Feb 2;24:311. doi: 10.1186/s12967-026-07775-1 (PMC12955024; doi:10.1186/s12967-026-07775-1)
Supplement: Supplementary file 2 — Supplementary Material 2 [file 12967_2026_7775_MOESM2_ESM.docx]

**Assessment and interpretation of statistical heterogeneity (I² Statistic)**

The I² statistic is calculated as I² = 100% × (Q − DF)/Q, where Q represents Cochran's heterogeneity statistic and DF denotes the degrees of freedom. Negative I² values are adjusted to zero, ensuring that I² ranges between 0% and 100%. An I² value of 0% indicates no observed heterogeneity, while higher values suggest increasing levels of variability. Specifically, I² values from 0% to 25% imply minimal heterogeneity, likely due to chance. Values between 25% and 50% suggest moderate heterogeneity, which may introduce some variability but generally does not significantly affect the interpretation of the results. An I² value between 50% and 75% indicates substantial heterogeneity, where considerable variability among studies could impact the reliability of the meta-analysis. Values exceeding 75% reflect considerable heterogeneity, implying that the meta-analysis results should be interpreted with caution due to significant variability potentially influencing the overall conclusions.
